# Regulation of filamentous phage Pf4 activation by oxidative stress in *Pseudomonas aeruginosa*

**DOI:** 10.1002/mlf2.70031

**Published:** 2025-08-25

**Authors:** Zixian Huang, Xinqiao Zhang, Shituan Lin, Jiayu Gu, Cong Liu, Mingzhang Wen, Yunxue Guo

**Affiliations:** ^1^ State Key Laboratory of Tropical Oceanography, South China Sea Institute of Oceanology Chinese Academy of Sciences Guangzhou China; ^2^ University of Chinese Academy of Sciences Beijing China; ^3^ School of Chemical Engineering and Technology Tianjin University Tianjin China; ^4^ Zhejiang Institute of Tianjin University (Shaoxing) Shaoxing China; ^5^ State Key Lab of Synthetic Biology Tianjin University Tianjin China

**Keywords:** biofilm, oxidative stress, prophage activation, *Pseudomonas aeruginosa*, regulation

## Abstract

The filamentous prophage Pf4 is activated to produce phage virions during *Pseudomonas aeruginosa* biofilm formation, a process crucial for maintaining biofilm architecture and enhancing pathogenicity. However, the environmental cues triggering Pf4 activation have been inadequately explored. In this study, we discovered that oxidative stress, a significant stressor encountered by pathogens in biofilms or within eukaryotic hosts, triggers the production of the filamentous phage Pf4 in *P. aeruginosa* MPAO1 through OxyR. Under oxidative stress, the expression of *oxyR* is induced, leading to increased OxyR binding to the promoter region of the Pf4 excisionase gene *xisF4*, thereby facilitating Pf4 prophage excision and virion production. Thus, our study elucidates a mechanism by which bacteria exploit cytotoxic oxidative stress as a potent stimulant to activate the filamentous phage Pf4 within biofilms.

## Impact statement

Filamentous Pfs in *P. aeruginosa* are pivotal for biofilm formation, virulence, antibiotic resistance, and evolution of host cells, and they also mediate horizontal gene transfer among different strains. The control of Pf activation in biofilms encompasses genetic modules from both the Pf prophages and the host. Biofilm formation is shaped by a blend of environmental stresses and components within the biofilm matrix, with Pf4 phages playing a significant role. In this study, we revealed that oxidative stress stimulates Pf4 activation through OxyR binding to the promoter region of its excisionase gene *xisF4*. These findings elucidate the interplay between genetic components and environmental stressors in coordinating Pf4 activation.

## INTRODUCTION

In response to challenging environmental conditions, bacteria in natural settings frequently adhere and aggregate to form biofilms on various types of surfaces instead of existing as free‐living (planktonic) cells^1^. Biofilm formation serves as a prevalent and effective survival mechanism that bacteria have developed to evade antibiotics and other stressors that can hinder cell growth or induce cell death^1^, ^2^, ^3^. The process of biofilm formation is a complex and intricately coordinated progression that unfolds through five distinct stages: reversible attachment, irreversible attachment, biofilm maturation (comprising maturation I and maturation II), and dispersion^4^. Throughout these stages, bacteria encounter a variety of external and internal environmental stressors, including oxidative stress, nutrient deprivation, the SOS response, and fluctuations in temperature^3^, ^5^, ^6^.

*Pseudomonas aeruginosa*, recognized as one of the most formidable opportunistic pathogens, is a prominent model in biofilm research. Biofilm formation is a key survival strategy used by this pathogen, contributing to enhanced antibiotic resistance (manifested as an increase in the minimum inhibitory concentration, MIC) or tolerance (surviving without altering the MIC) in the presence of high concentrations of antimicrobial agents. Additionally, biofilms facilitate increased virulence by enabling the pathogen to evade the host immune system^7^, ^8^. The *P. aeruginosa* MPAO1 strain hosts two co‐resident filamentous prophages Pf4 and Pf6^9^, ^10^, with the expression of Pf genes being among the most upregulated genes during biofilm formation^10^, ^11^. Subsequently, a significant release of Pf phages within the biofilm was observed, and these phages play crucial roles in various stages of biofilm development^9^, ^10^, ^12^. The presence of Pf phages interacts with host and microbial polymers, leading to the formation of liquid crystal structures that enhance biofilm adhesion, desiccation survival, and antibiotic tolerance^13^, ^14^, ^15^, ^16^, ^17^. Furthermore, Pf phages also participate in horizontal gene transfer, contributing to the pathogenicity of their hosts^18^, while the adaptation of higher competitive fitness mutants is common within biofilms^19^, ^20^.

The activation of Pf4 prophages is regulated by genetic elements, including the excisionase XisF4 encoded by Pf4, which stimulates Pf4 production; its expression is suppressed by the repressor Pf4r^21^. Additionally, the Pf4‐encoded toxin–antitoxin (TA) system PfiTA inhibits the activation of Pf phages by disrupting their excision and replication^22^. Furthermore, the Pf6‐encoded TA system, kinase–kinase–phosphatase (KKP), inhibits Pf phages by regulating the phosphorylation of the histone‐like nucleoid structuring protein (H‐NS)‐like nucleoid binding protein MvaU^10^, which is a host regulator controlling Pf4 activation^21^. The process of biofilm formation is intricately governed by a series of chemical components, including cAMP and c‐di‐GMP^23^, ^24^, ^25^, quorum‐sensing (QS) molecules^26^, extracellular polysaccharide components^27^, and the accumulation of metabolites^24^. A previous study showed that the c‐di‐GMP phosphodiesterase PipA promotes the activation of the Pf4 prophage^28^, and it has been observed that Pf4‐encoded genes inhibit QS signaling^29^, leading to the activation of Pf4^30^. Reactive nitrogen species have also been identified as activators of Pf4^31^. Moreover, while upregulation of Pf4‐encoded genes occurs under anaerobic conditions, no production of the Pf4 phage has been observed^32^. Therefore, a comprehensive investigation into the interplay of environmental stresses and dynamic genetic factors in regulating Pf activation within biofilms is essential.

Oxidative stress plays a pivotal role in shaping biofilm diversity and serves as a defense mechanism in bacteria by inducing the generation of reactive oxygen species to combat bacterial pathogens^5^, ^33^, ^34^. In our previous research, a significant activator, the excisionase XisF4 encoded by the prophage Pf4, was identified as controlling the lysis–lysogeny switch of Pf4 in *P. aeruginosa* ^21^. In this study, we discovered that oxidative stress is involved in the induction of the Pf4 prophage by regulating the expression of *xisF4*. Specifically, the oxidative stress regulator OxyR, a master regulator, induced early in biofilm formation, subsequently activates *xisF4* expression by binding to its promoter. Consequently, the internally generated oxidative stress during biofilm formation acts as a critical signal for enhancing biofilm strength through the activation of filamentous phage production.

## RESULTS

### Oxidative stress activates Pf4 prophages in both planktonic and biofilm‐growing cells

**Figure 1:**
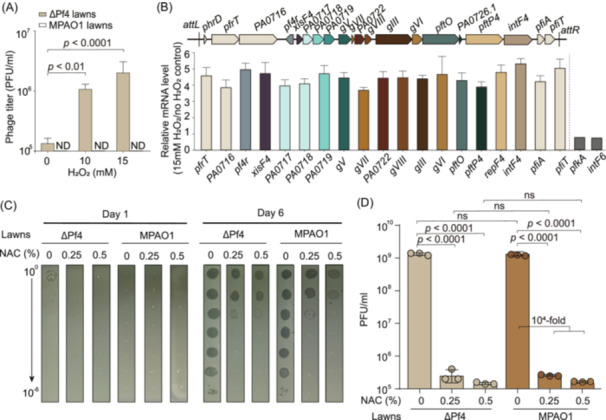
Oxidative stress induces the production of Pf4 phages. (A) Phage production of exponentially growing wild‐type MPAO1 cells (OD_600_ ~ 1.0) exposed to various concentrations of H_2_O_2_ for 30 min. The phage titers on both the MPAO1 and ΔPf4 lawns were calculated. “ND” denotes cases where phages were not detected. (B) The increased expression levels of the Pf4‐encoded genes but not the Pf6‐encoded genes when the MPAO1 cells were treated with 15 mM H_2_O_2_ for 30 min as shown in (A). Gene annotations for Pf4 are provided on the top. (C) Production of phages in MPAO1 biofilms inhibited by the antioxidant agent *N*‐acetylcysteine (NAC). MPAO1 cells were used for flow‐cell biofilm formation. Effluents were collected on Days 1 and 6, and serially diluted, and the diluted phages were plated on MPAO1 and ΔPf4 lawns, followed by incubation at 37°C for 16 h. Representative images were captured. (D) Phage titers on MPAO1 and ΔPf4 lawns calculated on Day 6. All experiments were conducted using three independent cultures, and the data presented in (A), (B), and (D) are indicated as the mean ± standard deviation (SD). “ns” indicates no significance. Only representative images are shown in C.

During the biofilm formation of *P. aeruginosa*, the filamentous phage Pf4 is activated, with oxidative stress posing a threat to bacterial cells within the biofilm^5^, ^12^. To investigate the role of oxidative stress in phage activation, we exposed planktonic MPAO1 cells to varying concentrations of H_2_O_2_ to induce oxidative stress. Following a 30‐min treatment, the phages released in the cultures were quantified on the bacterial lawns formed by the wild‐type MPAO1 strain and the ΔPf4 strain with the whole Pf4 prophage region removed from the host chromosome. The results revealed that increasing concentrations of H_2_O_2_ significantly increased the production of non‐superinfective (non‐SI) Pf phages from 1.3 × 10^5^ to 2.7 × 10^6^, as these phages infected only ΔPf4 cells rather than MPAO1 cells (Figure 1A). Since MPAO1 harbors both Pf4 and Pf6 phages, and phage production is linked to the expression of phage‐encoded genes^9^, ^10^, we further assessed the expression of these genes via quantitative real‐time reverse‐transcription PCR (qRT‐PCR) in MPAO1 cells treated with 15 mM H_2_O_2_. All the Pf4‐encoded genes were upregulated 4–5‐fold (Figure 1B), confirming that the produced phages are non‐SI Pf4, because the superinfective (SI) Pf4 lacks the region from *phrD* to the promoter of *xisF4* ^20^ and lacks the expression of *phrD*, *pfrT*, *PA0716,* and *pf4r*. However, non‐SI Pf4 contains this region and these genes are expressed. The expression of the selected Pf6‐encoded genes *pfkA* and *intF6* near the *attL* and *attR* sites of the Pf6 prophage, respectively, was not affected (Figure 1B).

We proceeded to investigate the influence of oxidative stress on phage production during biofilm formation by introducing the antioxidant agent *N*‐acetylcysteine (NAC) to scavenge the endogenous oxidative stress. The flow‐cell biofilm system, which induces endogenous oxidative stress over extended incubation periods, was utilized by supplementing 0, 0.25%, and 0.5% NAC in minimal, low‐osmolarity M9 medium (Figure 1C). As depicted in Figure 1C, minimal phage production was observed on Day 1 in the absence of NAC, whereas only a small number of non‐SI Pf phages were detected on ΔPf4 lawns rather than on MPAO1 wild‐type lawns. By Day 6, a large quantity of phages (~10^9^) was produced on both the ΔPf4 lawn and the MPAO1 lawn, predominantly comprising SI phages (Figure 1C,D). This phenomenon likely represents the reinfection and propagation of the genome‐reduced SI Pf4 phage variant, which lacks the lysis–lysogeny immune region, during biofilm development. The production of SI Pf4 is mediated by the reverse transcriptase PfrT in Pf4^20^. Notably, there was a substantial decrease in SI Pf4 phage production of approximately 10^4^‐fold on Day 6 with the inclusion of 0.25% or 0.5% NAC in the continuous flow medium (Figure 1C,D). Consequently, scavenging endogenous oxidative stress within biofilms leads to the suppression of Pf4 production.

### The activation of Pf4 phages in response to oxidative stress is regulated by OxyR

**Figure 2:**
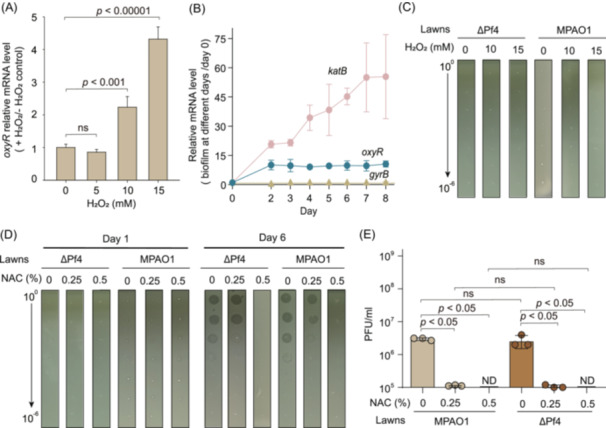
The activation of Pf4 phages by oxidative stress is *oxyR*‐dependent. (A) Transcript levels of *oxyR* induced by H_2_O_2_ treatment. Exponentially growing cells were exposed to various concentrations of H_2_O_2_ for 30 min, and 16S rRNA was used as an internal reference. Fold changes in *oxyR* mRNA levels at various H_2_O₂ concentrations were calculated as 2^−(CtoxyR+H2O2−Ct16SrRNA+H2O2)/2^−(CtoxyR−H2O2−Ct16SrRNA−H2O2). (B) Expression profile of *oxyR* induced during the early stage of MPAO1 biofilm formation. The expression level of *oxyR* was also normalized to that of 16S rRNA, and the fold changes were all calculated compared with those on Day 0. The expression levels of *katB* and *gyrB* were also determined as positive and negative controls, respectively. (C) No Pf4 prophage in Δ*oxyR* cells induced by H_2_O_2_ under planktonic conditions. Exponentially growing Δ*oxyR* cells (OD_600_ ~ 1.0) were exposed to 0, 10, and 15 mM H_2_O_2_ for 30 min. Phages in the cultures were serially diluted, and phage titers were determined on MPAO1 and ΔPf4 lawns. (D) Production of phages in Δ*oxyR* biofilms inhibited by NAC. Flow‐cell biofilm formation assays were carried out for 6 days using Δ*oxyR* cells. Effluents were collected on Days 1 and 6, and serially diluted, and the diluted phages were plated on MPAO1 and ΔPf4 lawns, followed by incubation at 37°C for 16 h. Representative images were captured. (E) Phage titers produced in Δ*oxyR* biofilms on MPAO1 and ΔPf4 lawns calculated on Day 6. The production of phages in Δ*oxyR* strain is approximately 1000‐fold lower than that of the wild‐type MPAO1 strain. “ND” denotes cases where phages were not detected. All experiments were conducted with three independent cultures, and the data presented in (A), (B), and (E) are shown as the mean ± SD. Only representative images are shown in (C) and (D). “ns” denotes non‐significance.

To elucidate how oxidative stress triggers Pf4 production, we examined the involvement of the key regulator, OxyR. The expression of the *oxyR* gene was assessed under various concentrations of H_2_O_2_ (0, 5, 10, and 15 mM) in planktonic cells, revealing significant upregulation of *oxyR* expression at 10 and 15 mM H_2_O_2_ in exponentially growing MPAO1 wild‐type cells (OD_600_ ~ 1.0) (Figure 2A). To further investigate the changes in *oxyR* expression within biofilms, where endogenous oxidative stress is present, we analyzed the dynamics of *oxyR* expression from Day 0 to Day 8. As shown in Figure 2B, *oxyR* expression increased 10‐fold on Day 2 compared to Day 0, coinciding with the initial attachment of cells to the catheter surface. This elevated expression level persisted from Day 2 through Day 8. As expected, the positive control *katB*, which is upregulated by OxyR under oxidative stress, showed consistent upregulation during biofilm formation. In contrast, the negative control, *gyrB*, maintained a stable expression level throughout the biofilm formation process (Figure 2B).

To explore the direct influence of OxyR on phage production, we initially assessed phage induction in planktonic cells exposed to various concentrations of H_2_O_2_ (0, 10, and 15 mM). In contrast to MPAO1 wild‐type cells, planktonic Δ*oxyR* cells did not show phage induction, as observed in both MPAO1 wild‐type and ΔPf4 lawns (Figures 1A and 2C), indicating OxyR‐dependent induction of Pf phages. In the context of biofilm formation via the flow‐cell biofilm setup, the Δ*oxyR* strain displayed a significant reduction in phage production, approximately 1000‐fold lower than that of the wild‐type MPAO1 strain (Figures 1C,D and 2D,E). Moreover, upon supplementing the M9 medium utilized for biofilm formation with 0.25% and 0.5% NAC, a notable impact on phage production in the Δ*oxyR* strain was evident. Specifically, the addition of 0.25% NAC resulted in a tenfold decrease in phage production, whereas 0.5% NAC completely inhibited Pf4 phage production in the Δ*oxyR* strain (Figure 2D,E). These findings suggest that other unidentified regulators may also be involved in the regulatory network controlling Pf4 activation in response to oxidative stress. These findings collectively support the conclusion that oxidative stress induces Pf4 production in a manner contingent on OxyR within the biofilm environment.

### OxyR upregulates *xisF4* expression by binding to its promoter region

**Figure 3:**
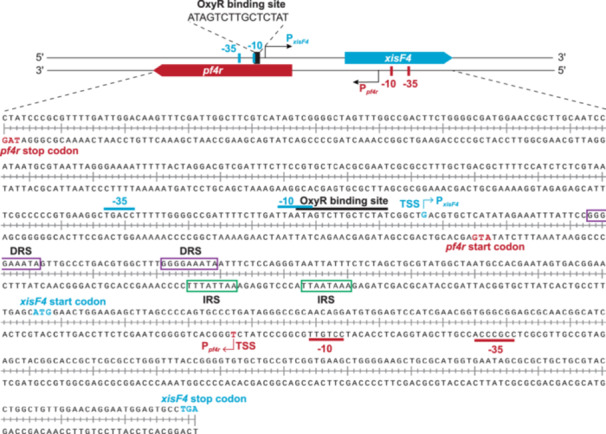
Sequence analysis of the *xisF4* promoter. In the *xisF4* promoter, the predicted OxyR binding site was identified (below the black line). The promoter regions of *xisF4* (blue) and *pf4r* (red) overlap, with “P” representing the promoter, and the potential ‐10 and ‐35 sequences are highlighted. Transcriptional start sites, start codons, and stop codons for both *xisF4* and *pf4r* are labeled accordingly. DRS and IRS denote the direct repeat sequence and the inverted repeat sequence, respectively.

*P. aeruginosa* controls oxidative stress through the H_2_O_2_‐responsive transactivator OxyR by binding to specific target sequences^35^. To explore the role of OxyR in Pf4 phage production, an online prediction database (https://www.prodoric.de/vfp/) was used to predict OxyR protein binding sites in the *xisF4* gene promoter. A potential binding site was identified within the *xisF4* promoter region (Figure 3). This binding site, 5′‐ATAGTCTTGCTCTAT‐3′, overlaps with the −10 consensus sequences crucial for transcription initiation. Furthermore, the *xisF4* promoter region was identified as a lysis–lysogeny switch that regulates the transition of Pf4 from the lysogenic state to the lytic state. This region contains a direct repeat sequence (DRS) and an inverted repeat sequence (IRS), which facilitate the binding of the H‐NS‐like nucleoid‐associated proteins MvaU and MvaT^10^, ^21^. These findings suggest that the regulation of *xisF4* expression is intricate, and involves the binding of multiple regulators.

**Figure 4:**
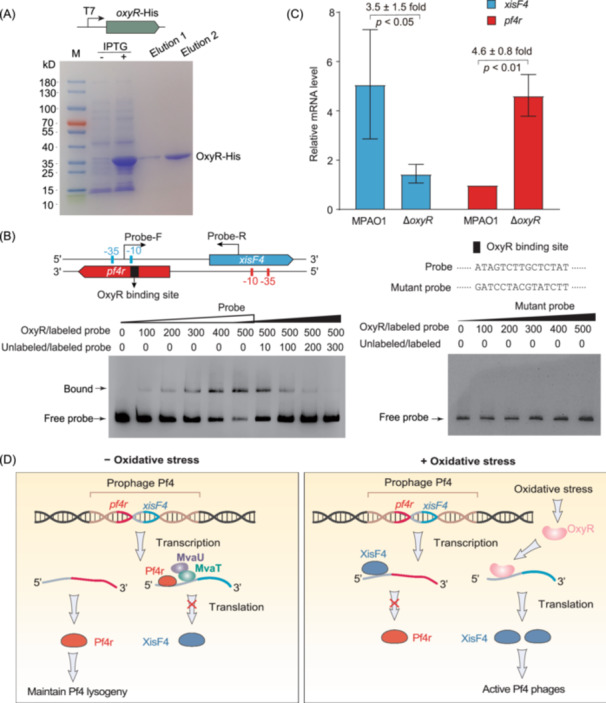
OxyR upregulates *xisF4* expression by binding to its promoter. (A) Purification of OxyR protein. C‐terminus His‐tagged OxyR was successfully isolated and purified, with the purified OxyR measuring 35.13 kDa in size. (B) Dose‐dependent binding of OxyR to the *xisF4* promoter region, with mutations in the binding site leading to the loss of OxyR binding. (C) The suppressed transcription of *xisF4* in the *oxyR* mutant strain and the upregulated expression of *pf4r*. Fold changes in *xisF4* or *pf4r* mRNA levels were calculated as 2^−(*C*t_*xisF4* or *pf4r*_ − *C*t_16S rRNA_)/2^−(*C*t_*pf4r* in MPAO1_ − *C*t_16S rRNA in MPAO1_). (D) Proposed model for the regulation of oxidative stress on Pf4 activation. Under normal growth conditions, *xisF4* and *pf4r* are transcribed under the control of their respective promoters. The expression of *xisF4* is repressed by Pf4r and H‐NS‐like nucleoid‐associated proteins MvaU/MvaT. The Pf4 prophage remains in a lysogeny state. However, during oxidative stress, the key regulator of oxidative stress, *oxyR*, is induced. OxyR binds to the promoter region of *xisF4* and enhances *xisF4* expression, subsequently inhibiting *pf4r* expression. The resulting XisF4 activates the excision and release of Pf4 phages. The experiment was performed with three independent cultures, and data are shown as mean ± SD.

To investigate the direct interaction of OxyR with the identified site, His‐tagged OxyR at the N‐terminus was purified (Figure 4A). A 313 bp fragment of the *xisF4* promoter was PCR‐amplified using MPAO1 genomic DNA as a template with the primer pair Probe‐F/R. The purified OxyR‐His and biotin‐labeled probes were utilized for electrophoretic mobility shift assay (EMSA). The results revealed a concentration‐dependent binding of OxyR to the promoter region (Figure 4B, left panel, lanes 1–6), with competition from unlabeled probes containing the same sequences as the biotin‐labeled probe used for OxyR binding (Figure 4B, left panel, lanes 7–10), indicating specific binding. To validate the specificity of the binding, the predicted binding site was mutated to 5′‐GATCCTACGTATCTT‐3′, and electrophoretic mobility shift assay (EMSA) under identical conditions demonstrated the abolishment of OxyR binding to the *xisF4* promoter region (Figure 4B, right panel). In conclusion, these results confirm that OxyR binds to the *xisF4* promoter region through a specific binding site.

**Figure 5:**
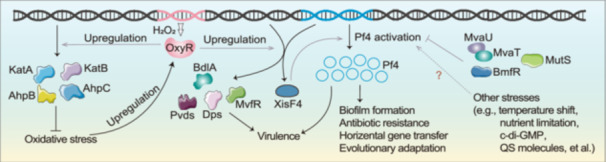
Proposed model for key OxyR regulation and potential environmental cues associated with Pf4 activation. The global transcriptional regulator OxyR is upregulated by H_2_O_2_, subsequently governing the expression of genes involved in oxidative stress and virulence in *Pseudomonas aeruginosa*. OxyR facilitates Pf4 activation by upregulating the excisionase gene *xisF4*. This induction process is also influenced by biofilm‐related environmental cues, although the precise mechanism remains largely unknown.

To investigate the influence of OxyR on *xisF4* expression, we evaluated *xisF4* expression levels in both the wild‐type MPAO1 and the Δ*oxyR* strains. As shown in Figure 4C, *xisF4* expression in wild‐type MPAO1 was 5.1 ± 2.2 times compared to *pf4r* in MPAO1, and 3.5 ± 1.5 fold compared to that in Δ*oxyR* during the exponential growth phase (OD_600_ ~ 1.0), indicating positive regulation of *xisF4* expression by OxyR. A previous study showed that XisF4 suppresses *pf4r* expression by binding to the DRS or IRS in its promoter region^21^. As expected, *pf4r* expression was elevated in the Δ*oxyR* strain (Figure 4C). Consequently, an oxidative stress model governs the fate of the Pf4 prophage by determining its transition between the lysogenic and lytic states (Figure 4D). Specifically, our findings suggest that in the absence of oxidative stress, the host regulator protein H‐NS and the Pf4 repressor Pf4r bind to the *xisF4* promoter region, repressing its expression and maintaining Pf4 in a prophage state. Conversely, under oxidative stress conditions, *oxyR* expression is induced, leading to OxyR binding to the *xisF4* promoter and subsequently enhancing *xisF4* expression while inhibiting *pf4r* expression. This coordinated regulation of *xisF4* and *pf4r* promotes Pf4 phage production within biofilms (Figure 5).

## DISCUSSION

*P. aeruginosa* forms in vivo biofilms upon infection of eukaryotic hosts, triggering responses from the host immune system and neutrophil cells to counteract this pathogen during the dynamic biofilm formation process^36^, ^37^. Among the array of strategies used, oxidative stress has emerged as a key mechanism by which accumulated reactive oxygen species target and eliminate bacterial cells. Our study elucidates how biofilm cells exploit oxidative stress to evade potential threats posed by eukaryotic hosts. The production of Pf4 phages confers several advantages to host cells, such as enhancing biofilm adhesion and structure, promoting antibiotic tolerance, enhancing virulence, preventing superinfection, and providing phage defense^9^, ^10^, ^12^, ^14^, ^38^.

OxyR, a LysR‐type transcriptional regulator (LTTR), functions as a redox‐sensitive transcriptional activator that upregulates antioxidant defense genes, such as *katA*, *katB*, *ahpB*, and the *ahpC–ahpF* operon, to combat oxidative^39^, ^40^. Furthermore, OxyR also activates other functional genes associated with the virulence of *P. aeruginosa*, including *dps*, *pvdS*, and *mvfR* ^35^. In this context, the identification of the Pf4 excisionase‐encoding gene *xisF4* was linked to binding in the −10 region, which was originally recognized as the promoter region of *PA0717* in Pf4 through ChIP‐chip analysis^35^. The binding of OxyR may facilitate RNA polymerase binding to this region, thereby increasing the expression of *xisF4*, which in turn promotes the excision and replication of Pf4 to generate virions. This activation mechanism could function as a bacterial defense strategy against oxidative stress. We demonstrated that the addition of H₂O₂ enhances Pf4 phage production in *P. aeruginosa* MPAO1 planktonic cells, generating phages capable of infecting only Pf4‐deficient strains. In contrast, prior studies showed that H₂O₂ activates Pf4 phages in planktonic cells that retain infectivity toward the original PAO1 host strain^31^. While the precise mechanism underlying this discrepancy remains unresolved, genetic and physiological variations among *P. aeruginosa* PAO1 sublines may contribute to these divergent outcomes^41^, ^42^. Given the widespread presence of Pf phages in ~50% of *P. aeruginosa* strains^43^, we propose that activating Pf prophages could provide selective advantages to bacterial hosts during infection.

Beyond the redox‐sensitive regulator OxyR, multiple host factors regulate Pf4 phage activation. For instance, H‐NS‐like nucleoid binding proteins MvaU/MvaT modulate Pf4 production via phosphorylation^10^, ^21^, ^44^, while the two‐component system BfmR controls Pf4‐associated gene expression through the antitoxin PhdA, a critical mediator of biofilm development^45^. Additionally, the DNA mismatch repair protein MutS suppresses Pf4 activation through an unknown mechanism^46^. However, the interplay between these regulatory pathways and environmental triggers remains poorly characterized.

Biofilm formation represents a collaborative group behavior demonstrated by cells in response to various stressors such as oxidative stress, the SOS response, temperature shifts, and nutrient depletion^24^. Furthermore, molecules such as cAMP and c‐di‐GMP^23^, ^24^, QS molecules^26^, extracellular polysaccharides^27^, and the accumulation of metabolites^24^ play important roles in biofilm development. Several findings suggest that these molecules are also involved in Pf4 phage activation^28^, ^29^, ^47^. Understanding the intricate interplay among environmental stimuli, chemical signals, and genetic components is essential for elucidating phage–host interactions within biofilms. This phenomenon is further exemplified in other bacteria, such as the prophage λSo induction in *Shewanella oneidensis* MR‐1 biofilms, which is required for extracellular DNA release and normal biofilm formation, and is suppressed by a low level of ions^48^. In addition, nutrient limitation promotes the activation of prophage CP4‐57 in *Escherichia coli* biofilms through the global regulator Hha^49^. Research on this interplay not only advances our comprehension of microbial behavior but also has significant implications for devising strategies to combat pathogens by targeting biofilm formation and phage activation mechanisms.

## MATERIALS AND METHODS

### Bacterial strains, plasmids, and growth conditions

The *P. aeruginosa* MPAO1 and ΔPf4 strains, as well as the conjugation donor strain *E. coli* WM3064, which requires 0.3 mM diaminopimelic acid (DAP) supplementation for growth, are maintained in the laboratory^10^. The primers used in this study are listed in Table S1. All the strains used were cultured in Luria Bertani broth (LB) at 37°C with agitation at 220 rpm. Gentamicin (50 µg/ml) and kanamycin (50 µg/ml) were used for plasmid maintenance (pEx18Gm‐up‐*oxyR*‐down and pET28b‐*oxyR*, respectively). A final concentration of 1 mM isopropyl‐β‐d‐thiogalactoside (IPTG) was utilized to induce *oxyR* expression via pET28b‐*oxyR*‐His.

### Total RNA isolation and qRT‐PCR

To assess gene expression in cells subjected to various concentrations of H_2_O_2_, overnight cultures of MPAO1 were diluted to an OD_600_ of 0.1 and grown until reaching 1.0. The cells were subsequently exposed to 5, 10, or 15 mM H_2_O_2_ for 30 min before being harvested for total RNA extraction. Total RNA isolation was carried out using a bacterial total RNA isolation kit (Tiangen). Following RNA quantification, 200 ng of total RNA was utilized to generate cDNA through reverse transcription using a kit from Promega following the manufacturer's instructions. The resulting cDNA was then diluted tenfold, with 1 µl used for qRT‐PCR analysis using the Step One Real‐Time PCR System from Applied Biosystems. The primers used for quantifying prophage and chromosome‐encoded genes are detailed in Table S1. The normalization of the gene expression data was achieved via the 16S rRNA gene transcript, and the fold change for each gene was calculated as previously described^50^, ^51^. Furthermore, the expression levels of *xisF4* and *pf4r* were also determined in wild‐type MPAO1 and Δ*oxyR* cells at an OD_600_ of 1.0.

### Flow‐cell biofilm assay

Flow‐cell biofilm assays were conducted following the detailed procedures outlined in our previous work^10^. To administer NAC treatment, 0.25% and 0.5% concentrations of NAC were supplemented in fresh M9 medium (47.8 mM Na_2_HPO_4_, 22 mM KH_2_PO_4_, 6.8 mM NH_4_Cl, 18.7 mM NaCl, 100 µM CaCl_2_, 2 mM MgSO_4_, and 0.1% glucose), with the medium being refreshed daily.

### Deletion of *oxyR*

For the deletion of the *oxyR* gene from the MPAO1 chromosome, a rapid and unmarked method was used following the protocol outlined in a previous study^52^. Initially, a 687 bp fragment upstream of *oxyR* and a 711 bp fragment downstream of *oxyR* were amplified using the primer pairs up‐F/R and down‐F/R, respectively, with genomic DNA from MPAO1 as the PCR template. Subsequently, the two fragments were purified via gel electrophoresis, and equal amounts of the purified PCR products were used as templates for a second round of PCR with the primer pair up‐F and down‐R. The resulting fused PCR products were also gel‐purified and quantified. Subsequently, 100 ng of the PCR product was ligated to pEX18Gm, which had been digested with *Sph*I and *Eco*RI, using the ClonExpress II One Step Cloning Kit. The ligation products were then introduced into WM3064 cells in LB medium supplemented with 0.3 mM DAP. Single colonies obtained were confirmed through PCR and subsequent DNA sequencing. The correct pEX18Gm‐up‐*oxyR*‐down construct in WM3064 was then transconjugated into MPAO1 cells for further selection of *oxyR* mutant colonies, following a previously described procedure^52^.

### Protein production and purification

The coding sequence of *oxyR*, with a His‐tag fused at the C‐terminus, was amplified with the primer pair pET28b‐*oxyR*‐His‐F/R and inserted into the expression vector pET28b, which had been digested with *Xba*I and *Hin*dIII restriction enzymes. The ligation and subsequent confirmation processes followed the same protocol as the construction of pEX18Gm‐up‐*oxyR*‐down. The OxyR protein, which was tagged with a hexahistidine at its C‐terminus, was purified from *E. coli* BL21/pET28b‐His‐*oxyR*. For protein expression, overnight cultures were diluted 100‐fold in fresh LB medium containing kanamycin (50 µg/ml) and 1 mM IPTG to achieve an OD_600_ of ~0.1. The diluted cultures were then incubated for 5 h. Subsequently, the cells were centrifuged and rinsed twice with phosphate‐buffered saline (PBS, pH 7.4), followed by suspension in lysis buffer containing 50 mM potassium phosphate buffer (pH 8.0), 300 mM NaCl, and a protease inhibitor cocktail (Sigma–Aldrich). Sonication was performed on ice for 5 min at level 2 using a sonic dismembrator from Ningbo Dongzhi, China. Subsequently, the cell lysate was centrifuged at 15,000 rpm for 1 h. The resulting supernatants were collected and stored at 4°C. Concurrently, Ni‐NTA resin (Qiagen) was equilibrated with lysis buffer three times and then incubated with the lysed supernatants on ice for 2 h to facilitate the binding of OxyR‐His to the Ni‐NTA beads. The binding mixtures were washed with various concentrations of imidazole ranging from 5 to 250 mM. Elutions with 200 and 250 mM imidazole were collected and analyzed using Tricine‐SDS‐PAGE. The purified proteins were desalted through disposable Sephadex G‐25 prepacked PD‐10 columns that were pre‐equilibrated with 20 mM Tris‐HCl buffer at pH 8.0, and OxyR concentrations were then assessed using the Bi Yuntian BCA assay kit (Haimen, Jiangsu).

### EMSA

EMSAs were conducted following previously described protocols^53^. Initially, a 313 bp region upstream of *xisF4* was PCR‐amplified using the primer pair probe‐F/R. Subsequently, the PCR products were purified from the gel using a gel extraction kit (Omega Bio‐Tek, Inc.). Then, 5 pmol of the purified fragments were labeled using the Pierce^TM^ Biotin 3′ End DNA Labeling kit (Thermo Fisher Scientific, Rockford, IL). The binding reaction was carried out with 0.1 pmol labeled probe, and increasing amounts of OxyR protein (0, 10, 20, 30, 40, and 50 pmol) in reaction buffer comprising 10 mM HEPES (pH 7.3), 20 mM KCl, 5% glycerol, 5 mM MgCl_2_, and NP‐40. In addition, to avoid the nonspecific binding between OxyR and the probe, competitor DNA (Poly‐di‐dC) was also added to the reaction mixture. The final volume of reaction is 20 µl. For the mutant probe with the OxyR binding site mutated, the same experimental conditions were used, with the exception that the OxyR binding site was mutated in the labeled probe. For the competition reaction, increasing amounts of unlabeled probe, containing the same sequence as the biotin‐labeled *xisF4* promoter, were added at 0, 1, 10, 20, and 30 pmol, together with 50 pmol OxyR protein, and 0.1 pmol labeled probe in the reaction mixture. The reaction mixture was incubated at 25°C for 1 h without agitation. Subsequently, equal volumes of each mixture were loaded onto a 6% DNA retardation gel (Invitrogen), transferred to a nylon membrane, and cross‐linked using UV light for 5 min. Chemiluminescence detection was performed using a LightShift Chemiluminescent EMSA Kit (Thermo Fisher Scientific) following the manufacturer's instructions.

### Statistical analysis

At least three independent biological replicates were utilized in all the experiments. Two‐sided paired Student's *t*‐tests were used to compare two groups with 95% confidence intervals. A significance level of *p* < 0.05 was considered statistically significant.

## AUTHOR CONTRIBUTIONS

**Zixian Huang:** Conceptualization; data curation; formal analysis; project administration; writing—review and editing. **Xinqiao Zhang:** Data curation; formal analysis; and methodology. **Shituan Lin:** Conceptualization; data curation; formal analysis. **Jiayu Gu:** Data curation; formal analysis. **Cong Liu:** Data curation; formal analysis. **Mingzhang Wen:** Project administration; supervision; writing—review and editing. **Yunxue Guo:** Conceptualization; data curation; funding acquisition; project administration; supervision; writing original draft; writing—review and editing.

## ETHICS STATEMENT

The study in this article did not involve any trials on humans or animals.

## CONFLICT OF INTERESTS

The authors declare no conflict of interest.

## Supporting information

SI.

## Data Availability

The data set generated during the study is available from the corresponding author upon reasonable request.
