# Supplementary material for: Regulation of filamentous phage Pf4 activation by oxidative stress in Pseudomonas aeruginosa
Source: mLife. 2025 Aug 25;4(4):437–46. doi: 10.1002/mlf2.70031 (PMC12395586; doi:10.1002/mlf2.70031)
Supplement: Supplementary file 1 — SI. [file MLF2-4-437-s001.pdf]

Supporting Information for
Regulation of Filamentous Phage Pf4 Activation by Oxidative Stress in *Pseudomonas aeruginosa*

Zixian Huang[1,2#], Xinqiao Zhang[3,4,5#], Shituan Lin[1,2], Jiayu Gu[1,2], Cong Liu[1,2], Mingzhang Wen[3,4,5*], Yunxue Guo [1,2*]

[1] State Key Laboratory of Tropical Oceanography, South China Sea institute of Oceanology, Guangzhou, China

[2] University of Chinese Academy of Sciences, Beijing, China

[3] School of Chemical Engineering and Technology, Tianjin University, Tianjin, China

[4] Zhejiang Institute of Tianjin University (Shaoxing), Shaoxing, China

[5] State Key Lab of Synthetic Biology, Tianjin University, Tianjin, China

[#] These authors contributed equally to this study

[*]Correspondence: Yunxue Guo, yunxueguo@scsio.ac.cn;
Mingzhang Wen, wenmz@tju.edu.cn

This PDF file incudes:

Table S1

Table S1. Oligonucleotides used for plasmid construction, DNA sequencing and qRT–PCR. F indicates the forward primer, and R indicates the reverse primer. "up" represents upstream region and "down" represents downstream region of *oxyR* ORF used for recombination during *oxyR* deletion. "M" in "Mprobe" represents mutant.

Primer Name	Sequence (5'-3')
**Cloning**	
pET28b-F	TAATACGACTCACTATAGGG
pET28b-R	TATGCTAGTTATTGCTCAG
pET28b-*oxyR*-His-F	CTAGTCTAGAGTTTAACTTTAAGAAGGAGATATAATGACCCTCACCGAACTGCGCTACA
pET28b-*oxyR*-His-R	CCCAAGCTTTTAGTGATGATGATGATGATGTGCTATTTGCGGTTGTTCCTGGGTTTGCG
pEX8Gm-F	AATCTTCTCTCATCCGCCAAAACA
pEX8Gm-R	CGCCCAATACGCAAACCGCCTCTC
up-F	GCCAGTGCCAAGCTTGCATGCGCGGCTCGCTGACCACCC
up-R	ACATGGCTGCTCATCCGTTAAG
down-F	TAACGGATGAGCAGCCATGTGACCGAGCTGTCCAGGGT
down-R	TATGACCATGATTACGAATTCGCTGCAGGGATAACTGGTGG
oxyR-conf-F	TCGACACCGATGTAGCAGTCCTC
oxyR-conf-R	CCTGGTAACCGGCCTCTATGGC
**Probe amplification**	
probe-F	GGCGATGGAACCGCTTGCAATCCATAATGCGTAATT
probe-R	GCTCATTCCGTCACTATTCGTGGCATTAG
Mprobe-F	CCGATTTTCTTGATTAGATCCTACGTATCTTCGGCTGACGTGCTCAT
Mprobe-R	ATGAGCACGTCAGCCGAAGATACGTAGGATCTAATCAAGAAAATCGG
**QRT–PCR**	
oxyR-qF	CTCGGTCGGCGTGAAGAA
oxyR-qR	TGCGGGAACAGGTAGGGA
pfrT-qF	GTTTTCGACTTCCCCTGCAA
pfrT-qR	CACACGACCCCAAACAAAGG
PA0716-qF	GGCGGGAGTAATGTGGTTGA
PA0716-qR	CCGGCCTTGTACTCTGCAAA
pf4r-qF	AGTTTGGCCGACTTCTGGG
pf4r-qR	CGTCAGCCGATAGAGCAAGA
xisF4-qF	CCGCAACAGGATGTGGAG
xisF4-qR	GCACTCCATTCCTGTTCCAA
PA0717-qF	TGCGCTACCTCGTAGAGATTTG
PA0717-qR	AGCGGCGAGCTTCTTCTTC
PA0718-qF	TCCGCAAGGCAGGTCATCC
PA0718-qR	TGGGCTTCCCGCTGTCGTAC
PA0719-qF	CACCCTTGAACGCCTCCC
PA0719-qR	CCTATCGCAACGTCGTCTCC
gV-qF	GGCGTCGTCGAACTGTGGGT
gV-qR	GCTGGCGAACTTGCAGAGCA
gVII -qF	ATGCTCCGCTATCTCTCGCTGTT
gVII-qR	TCAAACAGCCAGGGAGGCCGCTAGG
PA0722-qF	CGTTGTCGCTGTGCAGGTG

PA0722-qR	GCCAATCAAAAGCCCCGTC
gVIII-qF	GGTGGAATCGGCGATCAC
gVIII-qR	CGATGTAGCCGCCAATGG
gIII-qF	TTTGCGAGCCTGATTCTGATG
gIII-qR	GTCTGGTCACGGGCTTTCAT
gVI-qF	GCTCCAGACCCTGATCCTGC
gVI-qR	ACCTCCCAATGAACGGCAC
pftO-qF	CGGCAAGACCCAGGACACCA
pftO-qR	CAAGCCCAGGAGGAAGAAAG
pftP4-qF	GGTGGACGGTGATGACGG
pftP4-qR	CACCCCTACCGCCGCTA
repF-qF	GGCGTGCTGGATGATTTGG
rep-qR	GACAGTTGCAGGCCGTTGG
intF4-qF	AAAGTGGCTCGGTTGCGTAG
intF4-qR	AGTTCGGACACCTGATGCTTG
pfiA-qF	ATGCGGCTGACCTGGATT
pfiA-qR	TGAGCGAACCTCCTGGAAA
pfiT-qF	CACCACTTGGCTCCATTCC
pfiT-qR	CTGCTCAACGCTCTGCTTCT
pfkA-qF	CGACCTGAAACCAACAAACGT
pfkA-qR	TTAAACCATTGCTGAAAGGGAA
intF6-qF	GCTACTTGTCGGCGGTCTTC
intF6-qR	GATTCTTGGTCCGGTGATAGTG
16SRNA-qF	TGGTTCAGCAAGTTGGATGTG
16SRNA-qR	GTTTGCTCCCCACGCTTTC
